# The influence of peer mentoring on critical care nursing students’ learning outcomes

**DOI:** 10.1108/IJWHM-01-2018-0003

**Published:** 2018-06-04

**Authors:** Maureen Nokuthula Sibiya, Thembelihle Sylvia Patience Ngxongo, Somavathy Yvonne Beepat

**Affiliations:** 1 Faculty of Health Sciences, Durban University of Technology, Durban, South Africa; 2 Department of Nursing, Durban University of Technology, Durban, South Africa

**Keywords:** Competencies, Peer mentoring, Mentor, Critical care nursing, Critical care, KwaZulu-Natal

## Abstract

**Purpose:**

The purpose of this paper is to explore the influence of peer mentoring on critical care nursing students’ learning outcomes in critical care units.

**Design/methodology/approach:**

A qualitative exploratory research design was used to conduct the study. Ten critical care nursing students were recruited from critical care units in the five private and two public hospitals. Descriptions of their experiences were gained through individual face-to-face interviews.

**Findings:**

The study reinforces peer mentoring as a vital strategy in helping the critical care nursing students to attain their learning outcomes. However, peer mentoring was not consistent in all hospitals and there were no structured support systems to ensure that peer mentoring was formalized. Making peer mentoring a vital component in the registered nurses core competencies would enable efficiency and guarantee the viability of peer mentoring.

**Research limitations/implications:**

Mentors for the critical care nursing students were not included in the study.

**Practical implications:**

The study identified a need for incorporating a formalized mentorship programme into the core competencies of all qualified critical care nurses, the unit mentor to familiarise themselves with the prescribed learning objectives of the critical care nursing student and an allocation of supernumerary time for the critical care nursing student and mentors to allow for formal mentoring responsibilities to take place.

**Originality/value:**

The study reinforces peer mentoring as a vital strategy in helping the critical care nursing students to attain their learning outcomes and conscietises registered nurses of their responsibility as mentors.

## Introduction

Critical ill patients are at a high risk for actual or potential life-threatening health problems and therefore, require intensive and vigilant nursing care (Urden *et al.*, 2010). According to Sole *et al.* (2012), working in a critical care unit, either as a student or as a qualified staff member can be challenging. The critical care nurses of the twenty-first century are routinely caring for complex, critically ill patients, in a highly specialized environment (Morton and Fontaine, 2013). Critical care nurse training and education is designed to educate and train nurses working in a technologically advanced environment (Urden *et al.*, 2010). Critical care nurse training is made available to all registered nurses in the private and public colleges where training is offered at a diploma level with a duration of one year, and at the universities at a degree level with duration of two years. This training is registered with the regulatory body the South African Nursing Council (SANC) as critical care nursing (R2598 as amended by R260) (SANC, 1991). In order for critical care nursing students to coordinate and manage healthcare, maximum use of clinical teaching experiences need to be utilised in the critical care unit. The critical care nursing student must be able to skilfully deliver high-quality medical care, using all appropriate technologies. These students also have to follow stipulated teaching and learning outcomes that need to be achieved as per the SANC curriculum as detailed in regulation R212 as amended by No. R74 (SANC, 1997). Hence, peer mentoring is intended to be supportive in this stressful environment. Hunt and Ellison (2010) stated that peer mentoring provides opportunities for nursing students to build supportive student relationships.

## Background

Mentoring is promoted as a key strategy to support critical care nursing students. Mentoring in nursing can be traced right back to the pioneer of nursing, Florence Nightingale, who had more than one mentor and who in turn mentored others (Hurst and Koplin-Baucum, 2003). Mentors acting as peer teachers can ease the transition from the academic training classroom to the clinical unit by assisting the critical care nursing student with problem solving and clinical skills to be able to handle the emotional impact of the work. Peer mentoring means that students take responsibility for their own learning and active participation in their learning process. This helps them develop and enhance a range of skills including teamwork, collaboration, reflection and communication skills, which are important requirements in nursing (Christiansen and Jensen, 2008). Dracup and Bryan-Brown (2004) discovered that critical care nursing students are confronted with a vast array of complex situations and conditions, and a feeling of uncertainty and lack of confidence frequently surfaces. Clinical practice takes place according to the objectives of the curriculum and the training regulations of the Nursing Council (SANC, 1985). The overall objective is to provide learning opportunities to students, in every area of placement, based on the level of training so that they are competent at the end of their programme (SANC, 1985).

## Literature

Although the role of mentoring in nursing has been extensively researched, there is little research in South Africa, on peer mentoring of the critical care nursing student in the critical care environment and its relationship to their learning outcomes. Historically, nurses have supported each other in career advancement, and this has been done through mentorship (Luck *et al.*, 2017). As early as 1970, literature reported stress and anxiety as interrupting learning among nursing students in a clinical learning environment (Moscaritolo, 2009). This stress and anxiety is further compounded by advanced healthcare technology. When asked about common sources of anxiety, students most often mention: first experiences at a new clinical site, fear of making mistakes, concerns about performing clinical skills and using hospital equipment and lack of support from nursing personnel (Moscaritolo, 2009). Within the literature, peer mentoring appears to be a tool that plays a significant role in decreasing critical care nursing students’ anxiety and attrition in critical care areas (Buffum and Brandon, 2009; McGrath, 2009; Thomason, 2006).

Critical care nursing is a speciality that requires licensed professional nurses to be able to deliver competent care with confidence in the dynamic critical care environment where patients’ condition are unpredictable and can lead to life-threatening situations which need vigilant attention (Urden *et al.*, 2010). Characteristically, critical care nursing students lack the experience needed in order to apply the theoretical foundation received in their education, and hence seek the help of more experienced peers (Sims-Giddens *et al.*, 2010). A combination of senior students with less experienced peers can create a durable support system to aid in accomplishing the momentous task of preparing critical care nursing students for practice readiness and clinical competence in such situations (Ousey, 2009). Mentoring is seen as part of the socialisation of the nursing student into the nursing profession, where the mentor is a source of inspiration, and role model, forming a bridge between theory and practice, and this enables students to be fully functional once they qualify (Booyens, 2000; Warren, 2010).

Guillen (2010) questioned the reasons why peers were used to help ease the transition of the critical care nursing students from classroom into critical care settings instead of using nurse educators’ expertise. Guillen (2010) showed that there were numerous barriers to the concept of peer mentoring such as the severe shortage of nurse educators, hence making the ratio of available educators to students too great to enhance an effective mentoring relationship. Robinson and Niemer (2010) stated that there may be an increased understanding between both individuals promoting a more relevant learning experience and feelings of greater support and interaction by utilising a “student-to-student” approach to mentoring. Robinson and Niemer (2010) revealed that the demanding and uncompromising nature of the critical care nursing programme leads many critical care nursing students to discontinue their nursing education. Many critical care nursing students often underestimate the workload that the nursing programme entails, and try to balance many roles including family, work and personal health. The compounded stress of these multiple roles often leads to attrition (Robinson and Niemer, 2010). According to Hunt and Ellison (2010), the presence of peer mentoring programmes increases the retention rates and the chances of academic success for the critical care nursing student.

## Research aim

The aim of this study was to explore the influence of peer mentoring on critical care nursing students’ learning outcomes in critical care units in the province of KwaZulu-Natal (KZN).

### Research questions

RQ1. What influence did peer mentoring have on the critical care nursing students’ learning outcomes in the critical care unit?

RQ2. What were the experiences of critical care nursing students regarding mentorship in the critical care unit?

RQ3. What were the perceptions of critical care nursing students regarding mentorship in the critical care unit?

## Methods

A qualitative explorative research design was used to conduct the study guided by Benner’s novice to expert theoretical model. Benner introduced the concept that expert nurses develop skills and understanding of patient care gradually over time through the medium of a sound educational base as well as from numerous experiences (Benner, 1984). Benner describes five stages that the nurse progresses through from the time she/he enters the profession and or a new situation until she/he reaches full potential or expert status. These stages include novice, advanced beginner, competent, proficient and expert stage. Similarly, the critical healthcare nursing student entering the critical healthcare unit goes through the same steps in order for her/him to reach her/his full potential and independence in the critical care nursing profession. The critical healthcare nurse enters the critical care unit as a novice and is expected to exit as an expert in the field of critical care nursing. Through peer mentoring the critical care nurse is being guided to transit through the various steps of the Novice to Expert Model requiring a closer and detailed mentoring in the beginning then being gradually released and allowed more independent practice as she/he becomes more experienced. The critical care nursing students in the current study, who had already had been in the course for six months or more, described their experiences regarding peer mentoring in the critical care unit and the influence that peer mentoring had on their learning outcomes and their perceptions regarding mentorship in the critical care unit. The researcher used this information to gain deeper understanding (explore) the influence of peer mentoring on critical care nursing students’ learning outcomes in critical care units which was the aim of the study.

### Setting

The study was conducted on post basic critical care nursing students who were working in critical care units in both the private and the public healthcare sector in the province of KZN. For the purpose of this study, the private healthcare sector is referred to as Health Sector A and the public sector as Health Sector B in order to maintain confidentiality and anonymity.

### Sampling

A total of seven hospitals in which the critical care nursing students were being place for practical learning were purposively selected; five of which were from Health Sector A and two were from Health Sector B. A total of 10 consenting post basic critical care nursing students who were employed in the critical care units (four from Health Sector A and six from Health Sector B) and who were already in the course leading to the registration for a Diploma in Medical and Surgical nursing in critical care for six months or more were purposively selected. The sample size was guided by data saturation which was reached after eight interviews. Two additional interviews were conducted to confirm data saturation to give a total of ten interviews.

### Data collection

Data were collected using individual face-to-face in-depth semi-structured interviews with the participants between November 2014 and January 2015. The in-depth interviews were conducted by the researcher in English, with the use of an interview guide. The researcher had no personal relationship with the participants. The interview guide contained a demographic section as well as a central question to focus the discussion. The initial question that was asked was “What influence does peer mentoring have on the critical care nursing students’ learning outcomes in the critical care unit”. Probing questions were then used to elicit more information from the participants depending on their responses. Interviews were scheduled for 30 to 45 minutes for each participant. The interviews were recorded by audiotape to provide an unobtrusive and accurate record of the participant’s comments. Data were analysed using thematic analysis. Each interview session was analysed on the same day as the interview, before conducting the next interview, in order to monitor data saturation. The researcher moved from one study site to the next for the subsequent interview in order to ensure that participants from all seven hospitals were interviewed. Tesch’s open coding approach was used to analyse the data (Tesch, 1992).

### Trustworthiness

Methods of enhancing trustworthiness were utilised and the following four principles outlined by Lincoln and Guba’s strategies of credibility, transferability, dependability and confirmability were applied (Lincoln and Guba, 1985). Credibility was achieved through the accuracy of the description of the parameters of the study (who, where and when). Participants were purposively sampled and information was collected until data saturation was achieved. In order to enhance consistency, the researcher conducted a pre-test with two participant prior to the study. These participants did not participate in the main study. Two strategies to enhance transferability in this study were: data saturation, whereby additional participants were not providing new information, meaning that emerging themes became repetitive; and purposive sampling, where participants were purposefully selected in terms of the knowledge of the phenomenon under investigation (Brink *et al.*, 2012). To ensure that no bias influences the results, tape recordings were utilised, and these were preserved for further auditing, thereby, the perceptions of the participants would be reflected accurately.

### Ethical consideration

Before commencement of the study, ethical clearance was obtained from the Durban University of Technology Faculty Research Committee (REC 45/14). Data collection was only commenced after the KZN Department of Health and the managers for the participating hospitals had provided approval that the study be conducted in their facilities.

The researcher personally approached and addressed the critical care nursing students who were already in the prescribed course for six months or more. Information letters were also given to the prospective participants to read at their own time in order to gain more clarity about the study. The nature of the study, the right to refuse to participate, the risks as well as the benefits of the study were fully described to all the prospective participants and were also specified in the information letters. Participants were not obligated to be part in the study, and they were informed that they could withdraw from the study at any stage during the research process if they wished to do so. All the participants made an informed, voluntary decision to participate in the study. Written consent was obtained from all the participants who agreed to take part in the study. The hospitals and participants were coded so as to protect their identity.

## Findings

A total of ten participants were interviewed; four from Health Sector A and six from Health Sector B. All the ten participants were females, four were within the 26–30 age groups, and six were within the 31 years and above age group. With regard to duration of their experience in the critical care unit, all ten participants had between 6–18 months of experience as critical care nursing students.

The three major themes that emerged included the benefit of peer mentoring to learning, supervision of critical nursing students and experience in the critical care units. Several sub-themes aligned to each major theme emerged. The three themes and sub-themes are presented in Table I.

### Major theme 1: benefit of peer mentoring to learning

The majority of the participants highlighted the importance of peer mentoring in their learning and that the benefit of peer mentoring was that it compliments students’ competence level, results in autonomy and work independence, assures personal and professional growth of the critical care nursing students and increases self-esteem and confidence of the critical care nursing students.

#### Sub-theme1.1: complimenting students’ competence level

The participants verbalised that peer mentoring made them feel confident, and are able to function effectively as part of the team. They stated that receiving peer mentoring impacted positively on clinical competence and enabled attainment of some learning outcomes. “[…] . But, when we get peer mentoring, it really assists us because there is a lot of things we don’t understand […] ” (Participant 2, Health Sector B). “[…]. the peer mentors give us the information that we need, and they try to explain what is to be done in the unit, and what we expected to do […] and with their guidance we gradually become competent […] ” (Participant 1, Health Sector A).

#### Sub-theme 1.2: autonomy and work independency

Being mentored in the critical care unit has helped the participants to attain autonomy so that they are able to work independently in a confident manner. “[…] Critical care nursing students develop a sense of independence, after the peer mentor has worked with them […]. The student learns how to integrate theory and practice” (Participant 6, Health Sector B). “[…] I think peer mentoring is very good and it should be encouraged. It is the best way to ensure that the students develop autonomy and work independency […]” (Participant 1, Health Sector A).

#### Sub-theme 1.3: personal and professional growth

Participants stated that as much as the critical care nursing programme is a challenging one, they are in the process of development. Peer mentoring assisted them to grow professionally and personally “[…]. At the beginning I did not know what I was doing, but now with peer mentoring I have grown in my profession and became a stronger person […]” (Participant 6, Health Sector B). “[…]. At the beginning I was very nervous and with the assistance of people that supervise us I have grown professionally and have developed some confidence in performing my duties” (Participant 3, Health Sector A).

#### Sub-theme 1.4: increased self-esteem and confidence

Participants verbalized that due to peer mentoring, they are less afraid of the critical care environment; they feel confident within their training process. “[…]. The peer mentoring makes me confident and I am not afraid to do my work […] I am free to ask questions” (Participant 3, Health Sector A). “[…]. I do not think I would have managed without the support from my mentors, probable I would have left training. With their help I have developed self-esteem and confidence, now I can stand on my own and I am sure I would make a good nurse when I qualify” (Participant 1, Health Sector A).

### Major theme 2: supervision of the critical care nursing students

Some of the study participants were concerned about the supervision that was offered to them during practical. They stated that they were not supervised adequately either because there were no trained skilled staff to do supervision or the mentors were not available to supervise them as they had their own workload and responsibilities to carry out. Poor supervision was also associated by participants with reluctance of peer mentors (senior students) to supervise the juniors.

#### Sub-theme 2.1: shortage of trained skilled staff

Due to the shortage of trained skilled staff, critical care nursing students are expected to work as skilled trained staff in the critical care unit, without supervision. “[…]. Most of the time there is no one in the unit but just us as students. We are expected to function as if we are already critical care specialists. We are treated just like critical care sisters; we’re expected to do the work of the critical care sisters […]” (Participant 2, Health Sector B). “[…]. Most of the staff in the unit are the students with just one or two trained staff, it is really impossible for them to be able to give us enough attention because they need to prioritise the patients […]”. (Participant 5, Health Sector B) “[…]. The problem is there are very few trained staff in the critical care units and so we do not even worry about supervision and mentoring, our priority is taking care of the patients so we just work and pray all is well […]” (Participant 4, Health Sector A).

#### Sub-theme 2.2: workload and responsibilities for the mentors

Some of the participants verbalized that they were on their own without a mentor to supervise; in most cases the mentor had her own workload and responsibilities. “[…]. So my heart really goes out for the peer mentors; where they have their own patients and their own workload and responsibilities. While they have us to help they still have to worry about the patients and they have to prioritise the patients over us, it is their core responsibility” (Participant 5, Health Sector B). “[…]. Most of the time we are neglected because the same person that is allocated to do peer mentoring has to attend to patients at the same time […]” (Participant 2, Health Sector B).

#### Sub-theme 3: reluctance to mentor students

The participants stated that the trained registered nurses in the critical care unit were of the impression that it was not their responsibility to teach the critical care nursing students, but that of the critical care nurse educators “[…]. We as student are just the workforce, there is nobody to mentor or supervise you, even the registered nurses and doctors they also don’t make time to teach us, they feel it’s not their duty to teach anyway” (Participant 2, Health Sector B). “[…] .Sometimes you wonder whose duty it is to mentor us in the wards because the registered nurses in the wards always argue that it is not their duty whilst on the other hand our tutors are also not willing to accompany us […] perhaps the department should employ people that they are going to pay like preceptors or clinical facilitators for the critical care nurses […]” (Participant 1, Health Sector A).

#### Sub-theme 4: reluctance to share information with students

The participants stated that the experienced critical care nurses acting as peer mentors were reluctant to share information with them which further impacted negatively on their attainment of their learning outcomes. “[…]. I would expect that seniors guide and support us and help us to work through the cases allocated to us but most of the time they hide their information and are not willing to share it with us […]” (Participant 3, Health Sector A). “[…]. Ai for now it’s difficult because some of the groups especially those senior to us do not like to share information with us, sometimes even your own a group mates […] some just keep whatever they got for themselves […] ” (Participant 1, Health Sector A). “[…] Perhaps learning will be much easier and faster if our seniors, because they already have the information, could be willing to work with us. Not that we want to be spoon fed but at least to give us ideas as to what we are expected to do then we can build on that and find our way through” (Participant 2, Health Sector B).

### Major theme 3: placement in the critical care units

The participants commented about the way their subsequent placements in the various critical care units was done and how this influenced their learning. The two sub-themes that emerged included, differences between health institutions where critical care nursing students were allocated and how the allocation schedule for each critical care nursing student was structured. While some participants described these two elements as enhancing their learning others described the very same elements as hindering progress in their learning.

#### Sub-theme 3.1: differences between critical care nursing units based on where students are allocated

The participants stated that the critical care nursing students are placed in different critical care units in different healthcare institutions. Based on size, equipment available and type of patients admitted, the critical care units function differently. The participants stated that it is sometimes difficult to adjust when you are moved from the one unit to the next as equipment and procedures are different especially in the absence of peer mentoring and supervision. “[…]. Sometimes things are unfamiliar even if you have worked in the critical care unit for some time because the units differ so when you are moved to the new unit it is like you are a new student. In some unit they assist you but in others you are thrown into the deep end […]” (Participant 5, Health Sector B). “[…]. What is good is that the critical care units are not the same, this allows us an opportunity to start small while you are allocated in a smaller unit with less ill patients and less complicated equipment. By the time you are moved to the more complex unit at least you have been guided and mentored a bit. Especially because the mentors are usually very busy to attend to us and you are left to find your own way through […]” (Participant 4, Health Sector A).

#### Sub-theme 3.2: structuring of the allocation schedule for each critical care nursing student

The participants indicated that due to the nature, size and organisation of critical care units in different institutions, transition from a critical care from one hospital to the other was sometimes not easy. Where the units are smaller, with lesser ill patients, there is not much exposure to critical care nursing. This creates a problem when a student is subsequently allocated to a bigger unit with more ill patients. The mentors recognise the student as being more experienced in training and do not provide mentoring. “[…]. It is much better if you are made to start in a smaller and less busy unit then you are gradually moved to the more complex units as this will allow you to learn a more comfortable and safer way. Yet our teachers do not consider this they just put you wherever there is space available irrespective of your experience, it is so unfair especially because we do not get adequate support and guidance from our mentors”(Participant 1, Health Sector A). “[…]. What assisted me was the way my allocation was structured. In the beginning while I was still new I was allocated to work in a smaller unit and now that I am more senior I am made to work in bigger institution. This also eases the burden on my mentors because at least I am able to correlate theory with practice” (Participant 3, Health Sector A). “[…]. It is how allocation is done that compounds our problem, our teacher know there are huge problems with mentors so at least they should be very careful in how they structure the allocation. This will at least assist us and ease the burden on the mentors otherwise they become irritable with us […]” (Participant 6, Health Sector B).

## Discussion

In an attempt to highlight the influence of peer mentoring on students’ learning outcomes in the critical care unit the participants commented about the benefits of peer mentoring. With regard to their experiences and perceptions regarding peer mentorship in the critical care unit, they spoke about supervision of critical nursing students during clinical placement and how placement in the critical care setting influenced learning. These were identified as the three major themes that emerged from the interviews.

### The benefit of peer mentoring

According to the participants peer mentoring benefited them by complimenting their competence level, developing autonomy and work independence, allowing them personal and professional growth and increased self-esteem and confidence. Luhanga *et al.* (2010) attested to this stating that the competence achieved by student nurses from peer mentoring enables them to think critically and perform clinical skills efficiently. Critical care nursing students are overwhelmed by the roles and responsibilities required when working in the critical care unit (Proulx and Bourcier, 2008). Orland-Barak and Hasin (2010) emphasised that it is the mentor’s responsibility to support the mentee in becoming autonomous in life. Mentors set expectations and provide the mentees with support in working towards becoming an autonomous critical care nurse. This enables the students to air and address their anxieties (Pritchard and Gidman, 2012) which are usually brought about by fear of making mistakes and lack of support from nursing personnel. Kim *et al.* (2013) attested to the effectiveness of mentoring with reference to lowering of anxiety and improvement of academic performance, and satisfaction of nursing as a career. Students learn leadership attributes through observing those displayed by mentors; these attributes include communication skills, problem-solving and decision-making strategies (Ousey, 2009; Warren, 2010). Research has shown that the experience of being mentored instils values and qualities in nursing students (Pritchard and Gidman, 2012).

Student nurses need to experience a sense of belonging within the nursing team, to ensure that their self-image, confidence and motivation are boosted (Casey and Clark, 2011). Critical care nursing students perceive good mentoring as having a positive impact in building their self-confidence, and this enables them to be a competent critical care nurse within the critical care unit (Kelly and Ahern, 2009). Mentors support of students with difficulties encountered within the clinical environment increases the students’ self-esteem and helps them to be socialized into the clinical environment (Bulut *et al.*, 2010). Mentors play an important role in helping the student to be accepted and supported in the clinical setting which influences the student’s ability and motivation to engage in clinical learning opportunities (Pritchard and Gidman, 2012).

### Supervision of critical nursing students during clinical placement

The perception of some of the participants was that they were not getting sufficient supervision in the critical care units. This was attributed to unavailability of peer mentors, increased workload of trained staff and reluctance of senior student nurses to supervise the junior student nurses. The study highlighted the fact that the critical care nursing student had experienced a decreased amount of supervision, and this led to fear and anxiety levels being increased. According to Dennison (2010), mentorship is meant to be a relationship where skills or knowledge is exchanged from someone with more experience to one with less experience. Thus the importance of the mentors to have adequate knowledge and skill in critical care nursing. This is supported by Ousey (2009) describing the ideal mentor as a person who is familiar with the content to be taught, is passionate about the idea of helping another, and who will support, reflect with, encourage and respect the less experienced person during their clinical experience.

The theory component coupled with the clinical component proved to be an overwhelming experience in the short duration of the critical care nursing course. Students were expected to function as qualified critical care nurses but with knowledge and skill deficiencies, this was not feasible. Mentors are key to facilitating the critical care nursing student into the practice role so they can function effectively and with confidence.

It was evident in the study that the peer mentors experienced an increased amount of workload, and this impacted on their ability and availability to mentor. However, mentors had other demands which were brought about by their own day to day workload. Gopee (2011) confirmed this by saying that there is insufficient time for mentoring of students because of work commitments and increased workload. Waldock (2010) emphasised that teaching is an important function of the registered nurse, including the facilitation of student nurses’ learning in the clinical environment. However, Omansky (2010) argued that adjusted workloads do not necessarily occur in order to accommodate the effective process of mentoring. In contrary other authors attribute this inhibits the mentor’s ability to supervise to lack of an understanding of the goals and learning outcomes of the critical care nursing student (Omansky, 2010; Hurley and Snowden, 2008). However, O’Driscoll *et al.* (2009) supported both views stating that the increased workload that the mentors have to deal with leads to inadequate mentor preparation as a barrier to successful mentoring.

Furthermore, Ousey (2009) argued that in order for mentoring to be effective the mentor needs to create an environment conducive to learning, bearing in mind the critical care nursing student’s needs and requirements. An effective learning environment will involve the use of a multi-disciplinary team in the delivery of teaching and assessment of the teaching process, dedicated and committed staff who enable others to learn through a variety of processes and who have been prepared to undertake their roles as teachers and assessors (Shirani *et al.*, 2016).

### Experiences of critical care nursing students

The participants commented that in the absence of established peer mentoring they were or could be assisted by experience and exposure to the critical are units; moving from smaller less complex critical care units to larger and more complex ones. This supports Benner’s novice to expert model (Benner, 1984) that with more exposure there is less need for supervision and mentoring. The same principle applies with the structuring of the allocation schedule for each critical care nursing students; allowing the student to first work in a smaller less complex unknit before allocating to the bigger more complex unit. According to Pritchard and Gidman (2012), in the absence of mentoring, socialisation of the mentee into the clinical environment is an area of importance, as the work environment plays a major role in the mentees competence and confidence levels. This is supported by Bulut *et al.* (2010) who state that when mentors support students with difficulties encountered within the clinical environment, it increases the students’ self-esteem and helps them to be socialized into the clinical environment.

## Conclusion

The critical care nursing students that were interviewed reinforced the importance of peer mentoring as a vital component to help meet their learning outcomes, and promote their development into a proficient nurse practitioner. Participants believed that making peer mentoring a vital component in the already qualified registered nurses’ core competencies would enable efficiency and guarantee the viability of peer mentoring for further critical care nursing students.

### Limitations

The mentors were not included yet their contribution could have enriched the study.

No other researchers or coders have been used.

### Implications

#### Policy development and implementation

The researchers recommends that a formalized peer mentorship programme be incorporated into the core competencies of all qualified critical care nurses, and that this be reflected in their performance appraisal. This will motivate the registered nurse to fulfil her/his independent function as a teacher.

#### Nursing education

Formal support in the form of peer mentoring is crucial in assisting the critical care nursing student to achieve their learning outcomes. Therefore, a formalized mentorship programme should be incorporated into the core competencies of all qualified critical care nurses, and be reflected in their performance appraisal. This will motivate registered nurses who are unit mentors to fulfil their independent functions as teachers. The unit mentor should familiarise him/herself with the prescribed learning objectives of the critical care nursing student. This will enable the unit mentor to delegate appropriately so that learning outcomes are achieved by the mentee. Despite that the critical care nursing student practices within her/his specified scope of practice, absent or inadequate supervision over the critical care nurse can lead to medico legal implications as patients’ safety is questionable

#### Institutional management and practice

There should be an allocation of supernumerary time for the critical care nursing student and their mentor to allow time for formal peer mentoring responsibilities to take place away from the clinical area, to facilitate assessment and feedback, and to enhance consolidation. Ongoing structured mentorship meetings involving all parties in the mentorship programme and college staff are conducted. This will ensure a well-coordinated mentoring programme and align theoretical and clinical teaching and learning for critical care nursing students. Opportunities for training of prospective mentors should be made available in order to capacitate the mentors with the required skill and to keep them acquainted with the mentoring needs for critical nursing students.

## Figures and Tables

**Table I:** Overview of the themes and the sub-themes

Themes	Sub-themes
Benefit of peer mentoring to learning	Peer mentoring compliments students competence level
	Autonomy and work independence
	Personal and professional growth
	Increased self-esteem and confidence
Supervision of critical nursing students	Shortage of mentors to do supervision
	Workload and other responsibilities for the mentors
	Reluctance of senior students to mentor the junior students
	Reluctance of peer mentors to share information
Placement in the critical care units	Differences between critical care nursing units based on where students are allocated
	Structuring of the allocation for each critical care nursing student to the critical healthcare units
